# Clients’ experiences on North America’s first take-home injectable opioid agonist treatment (iOAT) program: a qualitative study

**DOI:** 10.1186/s12913-023-09558-6

**Published:** 2023-05-26

**Authors:** Eugenia Oviedo-Joekes, Sophia Dobischok, José Carvajal, Scott MacDonald, Cheryl McDermid, Piotr Klakowicz, Scott Harrison, Julie LaJeunesse, Nancy Chow, Murray Brown, Sam Gill, Martin Schechter

**Affiliations:** 1 Centre for Health Evaluation & Outcome Sciences, Providence Health Care, St. Paul’s Hospital, 575- 1081 Burrard St, Vancouver, BC V6Z 1Y6 Canada; 2 School of Population and Public Health, University of British Columbia, 2206 East Mall, Vancouver, BC V6T 1Z3 Canada; 3 Providence Health Care, Providence Crosstown Clinic, 84 West Hastings Street, Vancouver, BC V6B 1G6 Canada

**Keywords:** Injectable diacetylmorphine, Injectable hydromorphone, Take-home doses, Opioid use disorder, Person centered care

## Abstract

**Background:**

To support public health measures during the COVID-19 pandemic, oral opioid agonist treatment (OAT) take-home doses were expanded in Western countries with positive results. Injectable OAT (iOAT) take-home doses were previously not an eligible option, and were made available for the first time in several sites to align with public health measures. Building upon these temporary risk-mitigating guidelines, a clinic in Vancouver, BC continued to offer two of a possible three daily doses of take-home injectable medications to eligible clients. The present study explores the processes through which take-home iOAT doses impacted clients’ quality of life and continuity of care in real-life settings.

**Methods:**

Three rounds of semi-structured qualitative interviews were conducted over a period of seventeen months beginning in July 2021 with eleven participants receiving iOAT take-home doses at a community clinic in Vancouver, British Columbia. Interviews followed a topic guide that evolved iteratively in response to emerging lines of inquiry. Interviews were recorded, transcribed, and then coded using NVivo 1.6 using an interpretive description approach.

**Results:**

Participants reported that take-home doses granted them the freedom away from the clinic to have daily routines, form plans, and enjoy unstructured time. Participants appreciated the greater privacy, accessibility, and ability to engage in paid work. Furthermore, participants enjoyed greater autonomy to manage their medication and level of engagement with the clinic. These factors contributed to greater quality of life and continuity of care. Participants shared that their dose was too essential to divert and that they felt safe transporting and administering their medication off-site. In the future, all participants would like more accessible treatment such as access longer take-home prescriptions (e.g., one week), the ability to pick-up at different and convenient locations (e.g., community pharmacies), and a medication delivery service.

**Conclusions:**

Reducing the number of daily onsite injections from two or three to only one revealed the diversity of rich and nuanced needs that added flexibility and accessibility in iOAT can meet. Actions such as licencing diverse opioid medications/formulations, medication pick-up at community pharmacies, and a community of practice that supports clinical decisions are necessary to increase take-home iOAT accessibility.

**Supplementary Information:**

The online version contains supplementary material available at 10.1186/s12913-023-09558-6.

## Background

Opioid use disorder (OUD) continues to impose a heavy societal toll due to its chronicity and the adverse social, health, and legal problems it poses for individuals, communities, and healthcare systems worldwide [1, 2]. In North America, the toxic drug supply, wherein drugs of uncertain origins are laced with unknown doses of substances such as fentanyl and benzodiazepines, has resulted in an alarming increase in overdose deaths that have led some regions to declare public health emergencies [3, 4]. The COVID-19 pandemic allowed regulatory agencies to adapt OUD treatment guidelines to align with public health safety measures that minimize the spread of the virus. Some of these temporary measures were long-overdue in the path to offer person-centered addiction care, including increasing the number of days and number of clients allowed to take opioid agonist treatment (OAT) medications off-site to consume without direct supervision (i.e., take-home) [5].

Oral OAT with medications such as methadone, buprenorphine, or morphine engages and retains many OUD clients in care [6–8] and reduces overdose mortality risk [9, 10]. Treatment delivery varies widely across settings and medications [11, 12]. In North America and Europe, oral OAT clients typically consume their medication at outpatient programs, pharmacies, detox/residential programs, or hospitals on a daily (or near daily) basis under health care providers’ observation [12, 13]. Direct observation allows providers to promptly act in cases of drug effects, including but not limited to over-sedations and drug intolerances (e.g., overdose). In a limited number of settings, the clinical team has the discretion to provide clients with take-home doses for unobserved self-administration if, for example, they demonstrate clinical stability, the ability to safely store the medication outside the site, a series of negative urine drug tests, and sustained OAT adherence of typically two to three months (criteria vary by region) [14–16]. To accommodate public safety protocols during the COVID-19 pandemic, OAT programs worldwide had the opportunity to increase the availability and accessibility of take-home doses [17–19]. For example, certified methadone treatment programs in the United States received an exemption from the federal requirements which enabled them to provide up to 28 days of take-home doses [20]. The increase in accessibility was a long-awaited expansion of OAT care for clients and providers [21–23], as travelling to the clinic daily, especially long distances, may decrease engagement with, and retention in, OAT [24]. Several studies reported early successes with the expansion of take-home OAT, such as high rates of treatment retention and client satisfaction, and no significant increases in opioid-overdose events [25, 26].

To attract and retain into care the critical minority of clients for whom oral treatment is either undesirable or ineffective, injectable OAT (iOAT) with medications such as diacetylmorphine (i.e., pharmaceutical grade heroin) or hydromorphone (other medications such as fentanyl, buprenorphine, and methadone may become accessible) have been shown to be safe and effective in several clinical trials [27–29], as well as cost-effective [30, 31]. The injectable formulations offered in iOAT (diacetylmorphine and hydromorphone) are short-acting (i.e., a rapid increase and decrease in serum levels) full mu agonist opioids (approx. 3–6 h). Oral OAT is mostly offered in long-acting form, from 24 h (e.g., methadone, morphine, both full mu agonist opioids) to 36 to 46 h (buprenorphine, a partial agonist). While all these treatments are capable of managing cravings and withdrawal symptoms, they differ on other subjective effects and bioavailability (e.g., via injection or oral ingestion). Also, while opioids are very similar in their action profile, there is a high interindividual variability on response to treatment [32]. Currently, daily observed iOAT (e.g., not take-home) is available in Canada, the UK, Netherlands, Germany, Switzerland, and Denmark [33, 34]. Minimum eligibility for iOAT includes severe OUD and to be currently injecting opioids [14]. Nevertheless, iOAT has historically been accessed by clients with long histories of injection drug use, unstable housing, and prior OAT attempts [35]. iOAT is typically administered under strict protocols and regulations [33, 36, 37]. While it varies per setting, usually clients attend up to three in-person appointments at their iOAT site every single day for an observed injection. The in-person visits give opportunities for safety monitoring, therapeutic relationship building, and access to wraparound services (e.g., social workers, counselling, other medical care) [38]. Specifically, direct observation allows providers to promptly act in cases of drug effects, including but not limited to over-sedations and drug intolerances (e.g., overdose) [39]. These benefits are important for many clients, yet the demands of iOAT render it an extremely high-threshold treatment option [40, 41].

While clinical guidelines currently offer the possibility for OUD clients to receive take-home oral OAT medication, this option had not been considered viable for iOAT [42]. The addiction care system’s reluctance to provide take-home iOAT is largely driven by an abundance of caution about client safety (e.g., overdose) and medication diversion, which is defined as “the selling/trading, sharing or giving away of prescription medications to others” [43]. The evidence from take-home oral OAT, the only available analogue, remains inconclusive regarding these concerns [22]. Further, as with take-home oral OAT, take-home iOAT is only indicated for clients who have the physical and psychosocial capacity to handle the medication off-site. Experiences in the UK provided a glimpse of the possibilities of take-home iOAT, as injectable diacetylmorphine was exclusively prescribed in pharmacies as take-home until the 1990s when the confluence of increased international acceptance of oral medications, fears of diversion, political pressure from the United States, and lack of research on effectiveness led to a drastic decline in prescriptions [33, 44]. Presently, take-home iOAT doses are prescribed to a limited population of UK clients (usually people with long term chronic health problems) who demonstrate adequate stability, and the treatment is restricted to the geographic regions where there are prescribers who choose to offer the treatment [45, 46]. While the Swiss guidelines previously permitted prescribers to make exemptions based on clinical judgement, these exemptions were only granted in exceptional situations and under strict eligibility criteria [18]. The pressures of the COVID-19 pandemic eased Swiss guidelines so that a greater number of clients could access take-home oral/injectable diacetylmorphine to a maximum of seven daily take-home doses [47]. Temporary measures due to COVID-19 also made take-home iOAT available for the first time in North America to clients in Vancouver, BC who met eligibility criteria, such as at least six months on a stable therapeutic iOAT dose and the ability to safely transport and store the medication offsite. These initial experiences with take-home iOAT, while small in scale, showed promising benefits for clients and supported an expansion/continuation of the program [48].

After the COVID-19 emergency risk mitigating guidelines enabled take-home iOAT (not previously available) to be prescribed for the first time in Canada to align with public safety measures and social distancing protocols, some BC iOAT sites expanded on these precedents to continue prescribing up to two of a possible three daily doses of injectable medications to select clients after the temporary guidelines concluded. The present study explored the processes by which iOAT take-home doses impact iOAT clients’ quality of life and continuity of care in real life settings. Secondly, we investigated how access to this program can be maintained within the dynamics of the larger community and context (e.g. drug policy, stigma, safety concerns, etc.). The primary audiences for this paper are care providers and policymakers across services who are interested in innovative approaches to addiction care that can mitigate the ongoing dual public health crises of overdoses/drug poisoning and COVID-19. Results of this study provided a unique opportunity to speak directly to clients about their experiences and learn how take-home iOAT programs can be further adapted and expanded.

## Methods

### Setting, design, and participants

#### Setting

The present study is set at a community clinic in British Columbia (BC), Canada. This community clinic provides iOAT to approximately 110 clients each week and is staffed by full or part time professionals including nurses, physicians, pharmacists, social workers, dieticians, clinical assistants, outreach workers, and mental-health workers. Clients typically attend the iOAT site one to three times per day and administer their injectable medication (e.g., diacetylmorphine or hydromorphone) under the observation of healthcare professionals. Prior to COVID-19, injectable medications were not allowed outside the clinic premises. Co-prescriptions are also available (e.g., methadone, slow-release morphine, fentanyl patches). Oral medications that are co-prescribed can be eligible for take-home.

Clients undergo an initial three-day fast induction protocol to reach their individualized iOAT dose. However, prescriptions are based on recommended protocols with a maximum daily dose of 1000 mg of diacetylmorphine and 500 mg hydromorphone. Clients can access up to three doses per day with no more than 400 mg and 200 mg per dose of diacetylmorphine and hydromorphone respectively [49]. No past OAT experience is necessary for an iOAT prescription, although recommended eligibility criteria are that clients have severe and current OUD with injection opioids, are available to attend clinic up to three times daily, and have past experience with OAT.

The pilot take-home program reduced clients’ daily clinic visits from two or three down to one (depending on the client’s prescription). After their witnessed morning dose, clients picked-up one or two take-home doses from the onsite pharmacy. Clients could also pick up any requested health care supplies (e.g., needle tips, tourniquets, disinfectant wipes). Clients became part of the take-home pilot program on a first come, first serve basis by expressing their interest to the clinical staff (e.g., their prescribing physician) during any clinic visit. The prescribing physician then assessed clients’ eligibility based on several factors, including that the client: has 3–6 months experience on a stable therapeutic dose of iOAT; regularly attends their supervised dose appointments; has no recent history of post-dose complication; exhibits social, emotional, and cognitive stability based on the prescriber’s judgement; can safely transport and administer their dose off-site; and ultimately would benefit from this program. Final decisions about the prescription were made in consultation with the Clinical Coordinator and the Pharmacy Coordinator, and clients had follow-up consultations (first weekly, then monthly) for monitoring. At the time study initiated, 11 clients were receiving take-home doses at the community clinic. Client capacity was limited as it was a pilot program, and pharmacy resources could only accommodate a limited number of pre-filled take-home syringes. Our participants (*n* = 11) thus constituted the complete population of take-home iOAT clients.

#### Design and participants

This is a qualitative longitudinal study that involved three rounds of semi-structured interviews over a period of 17 months (July 2021 to November 2022). Between October of 2021 and February of 2022, the program was stopped due to regulatory barriers for reasons that were not disclosed to the public [50]. Although the program restarted in February 2022, we gave the participants space to readjust to the reconstituted program before we connected for the final interview round beginning in August 2022. Interviews were conducted with ten out of the eleven clients who received one to two doses of injectable diacetylmorphine (and one client received hydromorphone) to use outside the site.

### Data collection

Interviews were conducted at a research office located in the vicinity of the partnered community clinic. The first interview round occurred in July and August 2021. Ten out of eleven clients who received take-home doses of iOAT at the time of interviewing participated. Interviews were scheduled as close as possible to the client’s initial carry dose to gauge preliminary expectations. The second round of interviews was initiated in late October/early November of 2021, when the program was temporarily stopped. Eight out of eleven clients who received take-home doses of iOAT at the time of interviewing participated in this round. A final round of interviews occurred from August to November 2022 (approximately 6–8 months after their take-home dose service had been resumed). Nine out of ten clients who received iOAT carry doses participated in this round. To recruit participants, the prescribing physician at the community clinic referred clients through word of mouth and recruitment cards.

All interviews were recorded using two audio-recording devices. Audio files were stored in a secure server, and transcribed verbatim by an outside transcription service located in Canada. Interviews ranged in length from 20 to 60 min. All participants provided informed consent and were compensated for each interview at a rate of $30 per hour or fraction.

In the interview, clients were asked to share their experience and perspectives on take-home iOAT with an emphasis on how they have or would affect their quality of life and continuity of care. The research team developed an initial topic guide of open-ended, non-leading probes (e.g., What role does take-home iOAT play in your treatment? What could be done to make iOAT more accessible?) based on this research question. Crucially, the topic guide probed about potential negatives/challenges associated with take-home iOAT (e.g., What things are not as good/not good about iOAT carries vs. going to the clinic? In your view, is there anything that you felt like you gave up by attending the site less? Thinking of taking the medication outside the site, how did you feel about your personal safety, the safety of the medication, or the safety of others?) to ensure that participants had space to offer concerns or criticism (Additional file 1). However, interviews followed a semi-structured approach in which participants had significant autonomy over the direction and content of the conversations. The research assistants who conducted the interviews were trained to remain impartial to prevent any personal positionalities from impacting participant responses. The topic guides were adapted iteratively to reflect lines of inquiry raised in the previous interviews that related to the research questions.

### Coding and analysis

Coding and analysis followed an interpretive description framework [51]. Coding and preliminary analysis were done cross-sectionally concurrently with interviews to inform revisions to the interview guides. The transcribed data was then analyzed longitudinally at the end of third round interviews. Researchers independently coded the initial data then formed a consensus on a set of initial broad thematic codes (i.e., a preliminary coding framework) through an inductive descriptive process by which the research team reviewed the data from transcripts and added descriptive categories (codes) to segments of text [52]. A reflexive coding approach [53] allowed for emerging themes and lines of inquiry to be integrated into the topic guides and the codebook to inform future interviews, interview rounds, and rounds of coding. Analysis was conducted on NVivo 1.6.

To manage and analyze the data, we developed analytic building blocks that organized, described and synthesized data. These building blocks included the original verbatim transcripts; the coded datasets (i.e., all data coded cross-sectionally after each interview round), code summaries (i.e., thematic summaries which describe key findings within each top-code for each round), interviewer memos (i.e., detailed reflections on contextual circumstances and non-verbal cues), and thematic summaries developed from the initial descriptive code summaries to support specific longitudinal analysis [54]. Code summaries included the main ideas discussed by participants; any surprises in the data; areas where there was disagreement or contradictions in the data (if any); patterns (same or different); considerations for analysis; and illustrative quotes. The summaries were used to identify codes or themes that would benefit from a longitudinal perspective. The transcribed data and code summaries were then revisited focusing on the identified themes to develop specific longitudinal code summaries based on patterns or changes that occurred over the three rounds of data [54]. A final report based on these descriptive summaries was framed, and illustrative quotations from the transcripts were chosen to underpin selected themes. The final results were reviewed by members of our client advisory committee to confirm they accurately represented clients’ experiences.

### Positionality

Critical social theory informed all processes and outcomes of this research, particularly how our roles within institutions shape our social positions and identities in ways that might privilege us and marginalize others [55]. These positions/identities can consciously or influence decision-making processes and how we interpret information. Specifically, we relied on Foucault’s theories of power/knowledge relationships that manifest through roles enacted on the body [56]. The researchers acknowledge how their personal positions and identities have influenced these research activities with particular thought to the power dynamics implicit in our roles as representatives of institutions. In our ongoing effort to learn, unlearn, and engage in reflexivity, we cite these philosophical assumptions and how our lens of bias may have informed the interpretive process of coding and analysis [57].

## Results

**Fig. 1:**
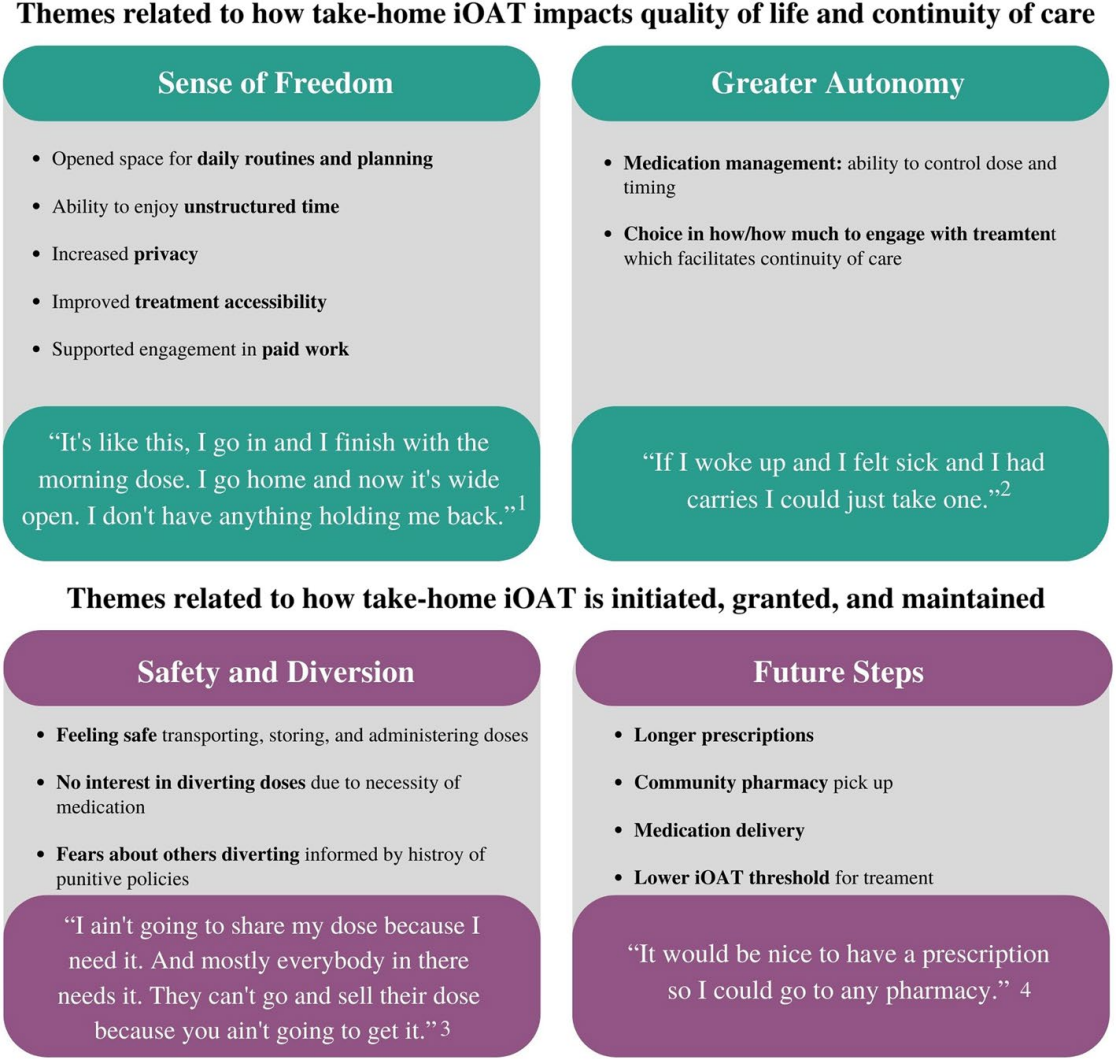
The four resultant themes from an interpretive description analysis of twenty-seven qualitative interviews with eleven participants ^1^ (Charlie, Round 2); ^2^ (Jessie, Round 1); ^3^ (Reese, Round 1); ^4^ (Peyton, Round 2)

Four broad themes emerged from the data, with associated sub-themes (Fig. 1). Two of these themes pertain to how reduced clinic visit impact quality of life and continuity of care: a sense of freedom, and greater autonomy. The other two themes pertain to how take-home iOAT are initiated, granted, and maintained: safety and diversion, and future steps for take-home iOAT. Specifically, participants connected a sense of freedom with their quality of life and continuation of care given the increased independence that attending the site once per day instead of three times afforded each of them. Their freedom from the iOAT site gave them more time to manage their day as they pleased, make short- and long-term plans, and work toward their self-identified goals. Increased quality of life and continuity of care were also achieved through a feeling of greater privacy, increased accessibility to treatment, and greater ability to engage in paid work. Participants gained the autonomy to manage their dose to align with their treatment goals, and this flexibility allowed them to consider long-term engagement in care. When take-home doses were temporarily disrupted, participants felt powerless and overwhelmed. Participants felt they could handle the medication well on their own and take measures to stay safe, although they acknowledged they cannot control what other clients do with their medication. Moving forward, participants see the program continuing to adapt to the needs of people that are doing well (e.g., as a reward) as well as becoming more accessible to those who are currently not being reached. Overall, participants expressed that the long overdue changes in the intensive structure of iOAT had a significant positive impact on their treatment and lives in general:

“*after the spending of these last few years and as stable as I have been, it’s not something that’s going to change my life negatively at all. It’s all positive. I can’t see anything but upside to carries.*” (Charlie, 1).

All participants were white, not currently experiencing homelessness, and born in Canada. All but two participants identified as male, and all but two participants identified with a disability as defined by the Accessible Canada Act (Table 1). Participants have been given aliases to protect their privacy. The number following the alias indicates which interview round the quote appeared in. As there are only two women and eight men, the aliases are gender neutral. While this paper uses the terminology “take-home iOAT,” many participants refer to the take-home doses as “carries”.

**Table 1:** Self-reported participant socio demographics^a^

**Participants**^a^	*N* = 10 (%); M ± SD
**Age**	54.71 (± 7.79)
**Years on iOAT** ^**b**^	10.86 (± 2.17)
**Type of Injectable Take-Home Medication**	
Diacetylmorphine	9 (90)
Hydromorphone	1 (10)
**Race**	
White/Caucasian	10 (100)
**Born in Canada**	10 (100)
**Gender**	
Female	2 (20)
Male	8 (80)
**Education**	
Up to Grade 7	1 (10)
Up to Grade 11	2 (20)
Some College/University	5 (50)
College/University Diploma	2 (20)
**Disabled**	8 (80)
**Housed**	10 (100)
**Partnered**	2 (20)
**Has Children**	6 (60)

^a^One client was part of the take-home program until his carry doses were reassessed and discontinued. He declined to be a part of this study

^b^Data only available for 7 participants

### Theme 1: sense of freedom, meeting a diversity of needs, and accomplishing self-identified goals

#### Daily routines and planning

Many participants felt receiving take-home iOAT was long overdue. Reducing their visits to the site from three (or two) to one brought, above all, temporal and spatial freedom from the site and the burdens inherent of attending multiple daily in-person clinic visits. While freedom from the site manifested differently for each participant, one participant described the practicality of take-home iOAT in this way:

*“Yeah. You get more freedom. I mean, strapped down to the clinic going three times a day for 15 years you have no – you can’t go – no time to go anywhere. You have to go there specifically so you’re not getting sick, right. If you want to go on vacation you don’t have your drugs you’re strapped down, you can’t go out of town. You can’t do nothing at work. So, you go in there and happen to – just playing off the freedom. Got more freedom, time to be able to do – have more of a life, you know, function, instead of knowing you have to come to the shop three times a day.”* (Sawyer, 1).

Interview round one occurred when participants were about to receive their first take-home dose(s). At this juncture, participants expressed optimism and excitement about the newfound time to carry out daily routines that had previously been restricted by their dose schedule and remaining in the vicinity of the clinic. These daily activities included anything from laundry and shopping to enjoying Sunday breakfast with their partner. By reclaiming these day-to-day spaces and activities while keeping their ideal medication, take-home iOAT supported participants’ quality of life. For example, a participant who cares for their daughter with chronic illness envisioned the upcoming time as follows:

*“Be home with my daughter and take her out for coffee in the morning and – she can’t go out without me, so her life’s gotten way smaller. Yeah. We could go to the beach, do things where we don’t have to stop in the middle of the day so I can go get my doses. Which she’s been doing with me for years. I drop her off at the coffee shop and I go – yeah”.* (Jude, 1)

Beyond their daily routines, participants began to make short and long-term plans for their time that had previously not been possible because *when you have to go [to the clinic] three times a day, you can’t go very far.* For example, one participant planned to *buy a motorhome and go travelling around the province* in this new era of mobility (Reese, 1).

#### Unstructured time

“*It’s like this, I go in and I finish with the morning dose. I go home and now it’s wide open. I don’t have anything holding me back”* (Charlie, 2).

In addition to being able to make plans, participants found joy in the temporal freedom and possibilities of an unstructured, open schedule with no set plans at all. One participant described how the freedom to flow in any direction on a given day ‘*increases my old sense of self-worth’* and allows them to be spontaneous and carefree with their time (Charlie, 1). For one participant, not having set daily plans access to take-home iOAT improved their quality of life by leaving space to attend spur of the moment outings with friends:

*“Yeah, I’ve got more of* [a social life] *because I don’t have to go to the clinic at times. It’s great! Like when people, “Ha, you wanna go take off? We’ll go camp out for the night?” Yeah! I can go do that. Go see a movie late at night. “Oh, well it’s going to start really early and that.” Oh, perfect. That’s fine by me. So it’s like I can’t – you know, I don’t have to limit myself to, “Well I can’t meet you until 9:30 because I gotta go to the clinic for 8:00” and things like that.”* (Jordan, 3).

#### Privacy

Prior to take-home iOAT, clients often had to wait on the street outside the partnered iOAT clinic before they received their dose. This experience was particularly common during the height of COVID-19 when the clinic operated at reduced capacity. Clients cited this as an invasive experience that publicly exposed folks who preferred to keep their affairs private. Participants shared that take-home iOAT gave them a greater sense of privacy because they did not have to make frequent clinic visits which exposed them to be *outed* to passersby (both strangers and community members they know). The sense of privacy also extended to clients’ work. Without take-home iOAT, clients who work during the day must leave the workplace, potentially more than once, to receive their dose(s). Clients are then put in an uncomfortable position where they must hide their absences if they prefer to maintain their privacy, or they must reveal their situation to an employer and risk being stigmatized.

“… *you’d be standing out there 10, 45 minutes waiting to get in to get your drugs and just completely outs you. Like, you have no privacy. And so like, having my coworkers and other people I see, see me there, like, you know, half the time they think I’m working there, and the other half they’re like, oh she’s getting her drugs. You know, like, it’s embarrassing*”. (Jessie, 1)

#### Accessibility

Participants cited both the time spent travelling to and from the clinic and the associated costs as major barriers to accessing iOAT, especially for participants who live far from the clinic and/or live with disabilities that impact their mobility or ability to leave the house. Making treatment contingent on getting to the site was seen as unfair, unsustainable, and as negatively impacting participants getting their ideal treatment. Participants expressed that take-home iOAT alleviated many of these accessibility issues by cutting a substantial amount of transit and cost out of their day.

*“Well I guess the big thing would be is, me, I live in [city outside of Vancouver], so it’s, you know, walking, busing, sky trains, you know. And then you know – and then having come back before – at first I started to come back maybe, you know, five or six hours later. And in the very beginning I did come three times, I switched it to two, so I’d only have to come here twice. And I guess maybe it took maybe a month to get used to twice, but that helped. I mean it just helped.*

*And it’s going to help, I know, a lot more just having to come here once, obviously, is the cost will be cut in half, and the time and it would free up more time for me to get back to work and be a more positive, more productive member of society.”* (Jamie, 1).

For other participants, having to come to an area with environmental triggers such as crowds, criminality, and drug paraphernalia incited anxiety and safety concerns that acted as a barrier to treatment. Take-home iOAT made treatment more accessible for these clients by reducing the amount of time spent in triggering environments.

#### Work

The population who accesses iOAT has historically been excluded from the labour market for reasons such as illness, disability, and stigma. For those who wanted to seek or increase their level of employment, the necessity of attending clinic visits during the work day restricted the scope of their work opportunities and shift scheduling. Take-home doses opened up participants’ schedules to engage in paid work and select shifts flexibly to fit their schedule: since accessing take-home iOAT, participants reported beginning new work roles, engaging in more work, returning to work, and/or taking courses to restart employment. Participants described increased self-esteem from being seen as reliable, accountable, important, and/or needed by their employers.

*Like when I had the carries my life improved greatly because I wasn’t like a ball and chain stuck to the clinic. I had more time away from the clinic. I could get better shifts at my volunteering and work and I just did well to not go in the clinics three times a day.* (Peyton, 2)

When take-home iOAT was temporarily suspended, participants felt frustrated that they no longer had freedom and flexibility with their employment. One participant shared that they now were forced to work night shifts, which is hard on the body and disrupts daily routines. These disruptions degrade quality of life or many prevent clients from engaging in paid work at all:

*“I can’t take shifts in the daytimes now, it’s all got to be night shifts. It sucks.”* (Quinn, 2).

### Theme 2: Autonomy sense of control over the management of the medication and engagement in care

The rigidity of the current iOAT guidelines places major limitations on clients’ autonomy. In the context of our interviews, autonomy fell into two categories: having autonomy over medication management, and having autonomy over level of care engagement.

#### Autonomy to make decisions to manage the doses and timing of the medication

Under the current approach to care, clients must take their dose according to the clinic’s operating hours and daily practices which reduces the control clients have over their medication management (e.g., a client who prefers to medicate in the evening may not be able if the clinic has already closed). With take-home iOAT, clients described an increased sense of autonomy over the timing of their medication which made them “feel a little more adult” and “not like we’re children” (Jessie, 1). Most participants reported that they changed their dosing habits or timing to align with their specific needs. For instance, clients valued their ability to medicate outside the hours of the clinic on a schedule that aligned with their body and alleviated discomfort:

*“I used to do it between 7:30 in the morning and 5:00 o’clock while I was getting my – before the carries. Now I can – that’s another beautiful thing about carries – I don’t have to do it all within a certain time of the clinic being open. I can take it later, even after the clinic’s closed, which is nice because that way I’m not antsy through the night. It carries me right through then.”* (Peyton, 3).

*“If I woke up and I felt sick and I had carries I could just take one. You know. And then I would feel instantly better and I wouldn’t have to wait that hour to get there feeling nauseous the whole way”* (Jessie, 1).

For the participants in this study, the main medication that meets their needs seems to be injectable diacetylmorphine. Combination or co-prescription with oral opioids is allowed and encouraged in a shared decision-making process with their prescribers [49, 58]. However, oral options such as methadone or morphine (available in our context for co-prescription with iOAT) were not viewed by the participants as a preferred medication that would allow them to comfortably visit the site once a day or less. Participants only briefly referred to the role of oral medications, particularly oral morphine, in the context of carrying them through the night. Participants who had temporarily switched to these oral treatments (e.g., to go on holiday) stated that these oral take-home doses did not meet their needs and at times have forced them to use illicit drugs to manage their withdrawal symptoms.

*“Well, the oral option just doesn’t work as well. For instance, I went to London a couple years ago. I was sick to my stomach for five days, till I could get used to [*oral morphine*]. That’s how weak it is, compared to the shots. I mean, violently sick. I got sick in the bed […]. It’s terrible. So it’s not a great option, but it’s all we have. And there is methadone, but I will never go back on that in my life. It was so hard to get off of. I didn’t sleep for 21 days.”* (Jude, 2).

iOAT clients can only access and inject their medications at the site where they received their prescription (i.e., they cannot go to other iOAT sites or community pharmacies). Some participants use co-prescribed oral morphine to cope with their work demands given the lack of pick up options for their injectable medication (i.e., pharmacy, other iOAT sites closer to their work sites) even if its not their preferred medication. For example, one participant expressed that going to the site three times per day was too cumbersome even though three doses would best meet his needs. Thus, clients may choose to visit the site for two injectable doses and take oral morphine co-prescribed. Even with the co-prescription, they will likely experience physical withdrawal symptoms in the morning and have to rush to the site. The take-home iOAT allowed participants to properly manage the timing of their doses and level of engagement with the site in a way that met their needs, showing that the role of co-prescribed oral medication among participants receiving injectables is supportive but limited.

*“Well I haven’t told* [prescribing physician] *that* [feels nauseous every morning*] because he’ll just say, ‘Oh, well let’s up you on the Kadian’ or, ‘Let’s get you another dose’ and I can’t come to the clinic three times a day, I don’t fucking have the time to do that. So I’m kind of stuck in this where I don’t know what I’m going to do now, like I’m on already 500 mg of Kadian. So I kind of found a sweet spot there where I was able to take it 12 hours apart* [referring to the time when they can manage the injectable themselves] *and it was working out perfect.[…] And I know if I tell the doctor that he’ll just be like, “Oh, well come in at 8:30 p.m. or nine o’clock” and it’s like I’m getting ready for fucking bed at that point, So I put up with that, I put up with being nauseous every morning because I don’t want to spend my whole life fucking going to the clinic.”* (Jessie, 2).

The autonomy that take-home iOAT provide was made poignant when the service was temporarily suspended in the Fall of 2021. During this time, clients reported feeling sad, frustrated, angry, resentment, upset, expendable, and let down by the addiction care system (e.g., policy makers, researchers, service providers). The reported loss of autonomy and control over their own health care evidences the powerlessness clients felt when they were one again bound by the schedule and routines of the clinic.

#### Continuation of care is supported by allowing autonomy in how clients engage with treatment

The current iOAT guidelines require that clients attend multiple daily in-person appointments. Participants expressed that the intensity of this approach to care makes it difficult to remain engaged or retained in treatment (i.e., going up to three times a day, every day). The rigor of iOAT even made some participants consider returning to the illicit market, particularly after benefitting from this service and being then temporarily suspended. Some found themselves at a juncture have to spend money and time on transportation, but at the same time not willing to risk their health by buying drugs in the street. Having the option to visit the site less frequently was seen by some participants as an incentive to remain in treatment long term during periods when they wanted to discontinue treatment:

*“I feel like before there was like I want to get out of this, I want to leave the program, and now I’m a little more content being in the program because I don’t have to go as much. Yeah I felt like when I was going twice a day I was just like I want to like taper off and get out of this, and like now I’m a little more content not having to spend that time every day. So my motivation for leaving the program has kind of dwindled because I’m more content with my treatment.”* (Jessie, 3).

Importantly, participants did not feel that having the option to engage with the site less frequently degraded the quality of their care. One participant indicated that coming once a day was more than enough to receive attentive healthcare:

*“No. I still have the same interactions with the nursing staff. I see them once a day is plenty. By that I mean if there’s any issues I need to bring up with respect to my physical health it can certainly be done then.* (Charlie, 1)

Offering the opportunity to engage with the site less frequently does not mean that clients will engage as little as possible; clients are welcome to maintain a higher level of involvement if it meets their needs (e.g., clients always have the option to come to the clinic more frequently than their one supervised dose). For example, some participants expressed a desire to remain be attached to the site on a daily basis for social connection:

*“I’m happy with the dailies, you know. I’m happy coming down here, you know. Because like I don’t have a lot of friends. So, you know, coming down here and socializing with [name] and [name] and the few people that I talk to in the program, you know. It’s pretty much the highlight of my day*” (Morgan, 1).

### Theme 3: safety and diversion

When clients begin the take-home program, they receive training from clinic staff on how to transport, administer, and manage their dose off-site. This training minimizes risk of diversion, lost doses, overdose, and injection related problems (e.g., infection). Clients also receive supplementary healthcare supplies. All clients interviewed had extensive experience injecting.

#### Safety

Once clients pick up their dose(s), they leave the site with the medication. This transportation and storage period might introduce potential opportunities for the medication to be lost, stolen, or diverted. Despite these possibilities, participants reported no concerns about transporting their dose from the site. Only one participant reflected on an initial anxiety that quickly dissipated as time passed:

*“At first I was a bit paranoid that somebody might grab my bag when I left [the clinic] because people know. But no, it hasn’t been an issue.”* (Jude, 3).

Part of the clients’ sense of safety during transport was that they were able to keep their dose private (e.g., in their purse, pocket, a lock-box provided by the clinic). Clients also took responsibility to be discreet about their take-home dose so as not to expose themselves to additional risk:

*“Oh God. It’s vitally important that people understand this. That medication should not be discussed with anybody. That medication is just medication for the client.”* (Charlie, 2).

Participants felt safe storing and administering their dose regardless of whether they live alone or with others (e.g., partner, children). Some would place their dose in a secure, hidden location that they were confident would not be found intentionally or accidently. Participants also reported that they felt extremely safe injecting because they were on a low or stable dose that they knew was right for them. Moreover, clients took overdose precautions such as having a NARCAN® kit available and injecting in the presence of others, for example, a participant expressed:

*“I would let somebody know hey I’m taking my shot and I do it with people around.”* (Peyton, 2).

#### Diversion

Clients’ views on diversion were heavily informed by their experience with addiction care’s punitive policies. Overall, participants reported no intention to divert their medication because their individualized dose is necessary for their care. Participants shared that selling their dose would not benefit them in any way because they would be left with an unmet need:

*“And I ain’t going to share my dose because I need it. And mostly everybody in there needs it. They can’t go and sell their dose because you ain’t going to get it.”* (Reese, 1).

While all clients had no interest in diverting their dose, many clients were fearful of what others might do with their medication because of the unintended repercussions that the system might impose on their own care. Clients felt that if anyone diverted their medication it would affect everyone’s access to take-home iOAT:

“*There’s always a fear that somebody will wreck it for the rest of us by trying to sell their dose or you know, that’s always – could be an issue.*” (Jude, 1).

Ultimately, participants felt that rather than address diversion situations with restrictions and punitive measures that take care away from all clients, each case needs to be addressed on an individual basis where the goal is to address unmet needs that may have led to diversion rather than view diversion as a nefarious act:

“*I think you just have to give people the benefit of the doubt until they make a mistake, and then you have to figure out a way to make it work for that person. Because not everybody’s – we don’t all have the same life.”* (Jude, 1).

### Theme 4: future steps: Program needs to evolve along clients’ needs

Participants were asked about how they envisioned the future of their treatment evolving alongside their ever-changing individual needs. Clients consistently reaffirmed their desire for greater treatment flexibility as the current system does not align with their vision of a world where they can have dreams, goals, and plans. Specifically, clients vocalized their desire for forms of service that would free up their life so they have space for the things that matter to them:

*“There you go. It does. And I’ll – it does, because of my being able to remove myself from the appointments and having to be somewhere on a daily – every single day, more than once. Gives me an opportunity to find ways to improve my spiritual life, ways to improve my physical life, and ways to improve my social life. And do I have all those answers? Do I know exactly what I need to do? No. I don’t. But I’m willing to learn. I’m willing to ask questions. And that’s all I can do. Right? If I can continue to doing that, I’m headed in the right direction. I’m confident.”* (Charlie, 3).

Longer take-home prescriptions (i.e., greater number of doses given to the client before needing to return to the clinic) were consistently the most common request, although clients varied in how long they wanted their prescription to be extended (e.g., a week, a month). Clients shared that an extended prescription would allow them to visit family out of province, go on holiday, or spend extra time taking care of dependants. At the very least, clients shared they want longer prescriptions on a case-by-case basis for special circumstances (e.g., sickness, holidays). These participant suggestions should be interpreted within the current context where clients must attend the site daily without exception.

*“And that’d be beautiful if we could get seven-day carries. That would mean I can go away for a week with the family and stuff like that, right?”* (Peyton, 3).

*“It’d be better I think if we only had to go there three times a week or two times a week or whatever. […] It would free me more up for work, other shifts and stuff. Now I can only work really a night shift from 4 till 12 then if I want to, I can work till 8. One morning, I got caught working until noon. That’s the problem sometimes”.* (Reese, 3)

Participants highlighted the role of community pharmacies in person centered addiction care. Picking up prescriptions at whatever pharmacy will add to their quality of life, as is convenient for them, wherever they may be. The flexibility to attend various pharmacies reduces the stress, costs, and time spent on travelling to a specific iOAT clinic:

“*Or, better still, it would be nice to have a prescription so I could go to any pharmacy. If I’m traveling around, we’re going traveling, I can take my prescription in, here’s my script for today. Say if I’m in Alberta or let’s not go that far. Say if I go to Prince George for a week I can go to a pharmacy there and go there’s my prescription for a couple of days for heroin. And you get it filled right? That would be the ultimate.”* (Peyton, 2).

The need for an outreach service that would transport the medication to their home has been expressed across the interviews to overcome ongoing and unique situations participants face. In their vision, they stated an outreach service was particularly necessary on a case-by-case basis to meet the needs of clients who are sick, taking care of others, or living with disabilities (e.g., mobility issues). For example, one client who cares for her daughter who has complex medical issues stated that:

*“There are some days I wish I didn’t have to get up and move at all if [child is] having a rough day. In that case somebody like an outreach nurse, they come and give you your shot, would be fantastic, yeah.”* (Jude, 3).

Considering that iOAT is a potential option for those currently unable to access any type of OUD care, one participant envisions a future where an iOAT outreach program meets the needs of those left behind by the program’s current regulatory limitations:

*“But I’d like it to be more flexible because like the building I work at we have 24 women and like 20 of them are opiate-dependent. They’re doing sex work for their opiate problem, and because [iOAT site] is so difficult to get into and it’s so high-barrier having to get there two or three times a day a lot of them even though it’s free drugs it’s too high-barrier for them and their chaotic life to access. And so I just wish that there was – if it was delivered like it is methadone I feel like it would be a lot easier for folks in my building to access it. And that would slow down on crime and sex work and a lot of the other things that are going on*,” (Jessie, 3).

## Discussion

In the present study with participants currently receiving take-home iOAT as part of a pilot program at a community clinic in Vancouver, British Columbia, we investigated how reducing the number of daily on-site clinic visits from two or three to only one impacts participants’ quality of life and continuity of care and how this program can be maintained. The 11 people that enrolled in the pilot program were long-time iOAT clients who met the take-home eligibility clinical consensus (mainly, the capacity to handle the medication off-site) [16, 22]. Reducing the burden on clients to attend the clinic up to three times per day exposed the diversity of rich and nuanced needs that flexibility in addiction care addresses and could further address if take-home iOAT is expanded. The sense of spatial and temporal freedom from the clinic impacted participants’ quality of life and continuation of care by opening space and time to incorporate daily routines, form plans, as well as lowering accessibility barriers, increasing work opportunities, and providing greater privacy. Further, take-home doses empowered participants with the autonomy to make decisions about their own care to better address their healthcare needs. Participants were confident they could handle the medication well independently and thus felt program delivery must continue evolving with their needs. There are also opportunities for further adaptations of this take-home program to reach currently untreated people for whom present iOAT restrictions are too high-threshold.

Participants stressed that take-home doses enabled their treatment to integrate into, rather than control, their lives in a way that meets their diverse, individualized needs. The necessity of take-home doses to lead a normal, fulfilling life has previously been expressed by clients seeking oral OAT [13, 59]. For example, while participants expressed different ways they wanted to plan/use the time they no longer had to spend attending clinic visits (e.g., spending time with loved ones, errands, being spontaneous, engaging in paid work), the common thread was that they had the freedom to allocate the time in whatever way aligned with their personal needs and goals, and that iOAT did not stand in the way of accomplishing those goals. Prior research similarly indicates that clients who receive longer prescriptions of take-home methadone appreciate their newfound recreation time and ability to engage in work opportunities [60].

Our work shows the nuanced day to day moments where autonomy (or lack thereof) was impacted by reducing the number of daily clinic visits, as participants now had more decision-making power to manage their dose. Specifically, greater autonomy manifested in participants’ ability to take their dose throughout the day at times that reduced their withdrawal symptoms and aligned with their bodies’ needs. Giving clients control over their care so that they can meet their individualized needs is consistent with the core principles of person-centered care [61]. Moreover, experiences were nuanced: participants reported that the less frequent clinic visits did not degrade their quality or continuity of care, yet many still want to be maintain a relationship with the clinic at their own pace. Similarly, when a methadone program in Ontario, Canada increased their provision of take-home doses, there was actually *less* treatment interruption and discontinuation [26]. Client autonomy is a core aspect of person-centered substance use care as part of shared-decision making [61]. With no regulatory support, the addiction care system struggles to align with best practices in medicine [62, 63] and does not fully embrace these principles [64]. For iOAT specifically, the stance on take-home doses has been rooted in fear of diversion, monitoring, and control rather than in empowerment, trust, and autonomy [65, 66]. To meet the diverse, ever-changing needs of iOAT clients, policies and regulations need to empower prescribers to provide person-centered care that adapts to individualized client needs [67, 68].

While there are documented adverse consequences to medication diversion [69] and an abundance of concern that clients will divert their dose or experience safety risks while transporting the dose [70], the clients in this study reported no intention to divert their dose because it is a necessary part of their care that meets a critical need. Participants also had no fears about their personal safety during transport because they could keep their dose discreet and store it in a secure location. The widespread belief that clients ultimately aim to divert their dose thus appears to stem from the system’s inherent distrust of OUD clients rather than empirical or anecdotal evidence [71, 72]. From an overdose risk standpoint, the adverse outcomes for a client who diverts their dose are no worse than if that client had not been engaged in care and were left to rely on the illicit market [73]. It is unrealistic for the system to expect that a degree of diversion will not occur when access to take-home iOAT expands [72]. Rather than let the possibility of diversion lead to restrictive access and severe systems of control, a client-centered approach would be to understand clients’ unmet needs that lead to diversion (e.g., financial pressure, friends and family in need) and address them [74, 75]. It is also important to understand that while the system views diversion as a nefarious and threatening act, clients may view it as harm reduction and mutual aid (e.g., giving their dose to a friend in need) [73, 76]. Ultimately, participants shared that it is unfair and unrealistic to punish clients collectively for instances of medication mishandling rather than address those situations on an individual case-by-case basis. The current system engenders a climate of tension and surveillance wherein clients feel the need to monitor their peers so that no one ‘ruins it’ and gets treatment access revoked. While providers do express liability concerns about take-home doses [23], the healthcare system has historically marginalized OUD clients and now has an opportunity to increase access.

Participants recognized that their treatment needs will evolve over time (e.g., aging, personal growth, social changes) and feel that iOAT has not evolved alongside them to become more accessible. This lack of flexibility affects both current and potential new clients. For example, they describe take-home doses as a form of equity and accessibility in their treatment, as the time and expense of getting to the clinic multiple times a day was a major challenge to remain engaged in care, similar to prior research on methadone [24]. Prior research indeed indicates that the intensity of iOAT (e.g., multiple daily in-person appointments) is a deterrent for potential clients to want to engage in the program [77, 78]. Not surprisingly, participants reported that the demanding schedule of appointments made them consider returning to the illicit market, which aligns with previous research shows that clients will return to the illicit market if treatment is not accessible [79]. Travelling back and forth to the clinic multiple times a day is particularly problematic given that populations with OUD are more likely to experience disabilities (e.g., musculoskeletal diseases, chronic pain, depression) that might hinder their ability to physically make it to multiple daily in-person appointments [80, 81]. In the same way that oral methadone clients expressed they would be more likely to remain in treatment if take-home doses were expanded, participants viewed take-home iOAT as an attractive feature of the iOAT program that can encourage folks to remain in or engage with treatment [13].

Participants affirmed their optimism for greater treatment flexibility as the current system does not align with their vision of a world where they can have dreams, goals, and plans. They suggested evolutions in treatment such as longer prescriptions, the ability to pick up medications at community pharmacies, and an outreach medication delivery service. While these increases in accessibility are a large departure from the current rigid structure of iOAT, these forms of service delivery are already available for oral OAT medication [14–16]. Thus, participants vision for the future of their care is well within the system’s realm of capability to provide. IOAT program managers can incentive engagement into care with take-home options that meet clients’ accessibility needs (e.g., chronic illness, care-giving responsibilities, work commitments). Policy reforms, such as allowing more than two doses outside the site, will align with practices in oral OAT, and expand further beyond.

As of May 2023, clients remain able to access take-home iOAT at this community clinic. Due to enduring system-level regulatory barriers and clinic constraints, the program has largely remained stagnant in terms of client numbers and program structure (e.g., a limited number of clients continue to be able to access two of a possible three daily doses as take-homes, with one clinically supervised dose daily). By integrating existing and emerging evidence on unobserved doses from clients and providers perspectives into future iterations of the take-home iOAT program [20, 59, 82, 83], the addiction care system can provide effective, accessible OUD care to clients with diverse needs at both the current and upcoming sites. While take-home iOAT might not fit all clients’ circumstances, person-centered OUD care and shared decision-making implies that some form of take-home iOAT is an option for the client and provider to explore when it best meets the clients’ needs. Necessary future research includes further investigating providers’ perspectives on how take-home iOAT can expand to meet clients’ needs [22, 84], and further examining treatment accessibility for clients with diverse needs across broader geographic locations.

### Limitations

It is of note that almost all of the take-home iOAT participants were white men despite approximately a third of the potentially eligible iOAT client population at this community clinic being self-reported Indigenous and/or women [85]. The lack of diversity is important, as it speaks to certain barriers that might have prevented clients with diverse needs from requesting take-homes and/or meeting the eligibility criteria (e.g., uncertain housing situation, unmanaged anxiety). The clinical team reflects that there is still much to do to engage clients of diverse races who would benefit from take-home iOAT access. Lessons from this pilot study provide a starting point to engage in those reflections, specifically on what accessibility, equity, and diversity means for the provision of individualized iOAT care.

## Conclusions

The present study interviewed participants receiving treatment in a first of its kind take-home iOAT pilot program. Clients were mostly white men with an average age of 54.71 years who have been receiving iOAT under direct observation for an average of 10.86 years at the same site. Being able to take two of a possible three daily doses off-site allowed participants greater freedom and autonomy to manage their treatment and daily lives. They reported no safety concerns or intention to divert the medication, as they need their doses daily. Regulatory bodies must acknowledge that providers require the appropriate tools to offer person-centered care, and having take-home iOAT as an option increases the latitude for providers and clients to make collaborative decisions. While this study does not allow us to make conclusions regarding the safety or effectiveness of take-home iOAT, our results highlight the impact that increasing treatment flexibility can have on peoples’ lives in the nuance that can only be captured by qualitative methodologies. Given the accomplishments of the pilot and clients’ desire for even fewer restrictions, possible recommendations moving forward include increased availability of formulations (vials) that are accessible for single-dose use, medication pick-up at community pharmacies, delivery systems for those with accessibility barriers, and adaptations to guidelines that support clinical decision-making between providers and clients.

## Supplementary Information

**Additional file 1.**

## Data Availability

The interview transcripts have not been made publicly available as this would compromise participants’ confidentiality, but they are available from the corresponding author upon reasonable request.

## Abbreviations

OUD: Opioid use disorder
OAT: Opioid agonist treatment
iOAT: injectable opioid agonist treatment
PCC: Patient centered cared
