# Supplementary material for: Clients’ experiences on North America’s first take-home injectable opioid agonist treatment (iOAT) program: a qualitative study
Source: BMC Health Serv Res. 2023 May 26;23:553. doi: 10.1186/s12913-023-09558-6 (PMC10215060; doi:10.1186/s12913-023-09558-6)
Supplement: Supplementary file 1 — Additional file 1. [file 12913_2023_9558_MOESM1_ESM.docx]

**iOAT Carries Interview Guide**

**Service User Interviews**

**Introduction:**

Thank you for agreeing to participate in this interview. We are interested in learning about your perspectives and experiences regarding how injectable opioid agonist treatment (iOAT) carry doses were or are provided and impact your quality of life and continuity of care. Your feedback will help us to understand how iOAT carries programs could be improved and adapted to meet the diverse needs that people may have.

We also want to take this moment to explain about the outcomes of these interviews. As researchers, we are interested in hearing about iOAT carries, including whether the ways it is or was provided have worked or not worked for you and the motivations and processes around these outcomes. We will also be asking you about changes that would make iOAT carries meet your needs better. That said, as researchers we do not hold the power to change policy. We aren’t policy makers or government officials so while we hope that this study will help policy makers and other officials design iOAT carries programs that best meet your needs, we can’t guarantee that it will. We will make every effort to pass all we learn from this study to those who are in a position to implement changes.

You can talk as much or as little about your personal experiences as you’d like. We understand that decisions about healthcare can be sensitive subjects for many people. You have the right not to answer any questions and you can leave or take a break from the interview at any time without giving a reason.

This conversation is confidential to those present, as per our guidelines. We would like to record this conversation and transcribe it so that we can listen to it and ensure our interpretations are accurate. Only the research team will have access to the recordings and transcriptions, which are uploaded directly to a secure server at CHEOS, without names that can identify you.

**START RECORDING**

**Questions for Service Users:**

**Note:** These are broad questions that as the theory evolve will become more specific (iterations within the grounded theory approach). Probes refer to examples of areas to explore base to the study research question**.**

- What led you to want/receive iOAT carries? PROBE: Did you have a specific need you needed to address? Was there an event that brought you to it?
- How do you feel about getting iOAT carries? PROBE: Benefits? Challenges?
- Is there anything you feel like you are giving up by receiving iOAT carries? PROBE: Social or community connection? Lifestyle routine?
- What role do iOAT carries play in your treatment? PROBE: for long time? Transitioning to other treatments?
- What things do you prefer about iOAT carries compared to going to the site more frequently? PROBE: What things are not as good/not good about iOAT carries vs. going to the clinic? Can you think of an example on how being connected to the clinic less frequently is helping you in your treatment plans?
- What could be done to make iOAT carries more accessible? PROBE: Length of iOAT carries prescription? Ease of getting iOAT carries? More flexibility? Support?
- Are there any concerns you have around taking carries with you or the to place you live? Probes: Safety? What can be done to support you? Can you provide with examples of times where you did feel concerned?
- Are there any characteristics about you that you think impacted the way you received (or did not) iOAT carries?

[Once the interview is completed, and there are no other areas to explore or the time has run out, we thank the participants for their involvement. Provide an opportunity for participants to ask questions. Stop the recording. Provide participants needing emotional support with the contact information of counseling services.]
